# Magnetic Hydroxyapatite Bone Substitutes to Enhance Tissue Regeneration: Evaluation *In Vitro* Using Osteoblast-Like Cells and *In Vivo* in a Bone Defect

**DOI:** 10.1371/journal.pone.0038710

**Published:** 2012-06-07

**Authors:** Silvia Panseri, Carla Cunha, Teresa D'Alessandro, Monica Sandri, Alessandro Russo, Gianluca Giavaresi, Maurilio Marcacci, Clark T. Hung, Anna Tampieri

**Affiliations:** 1 Laboratory of Biomechanics and Technology Innovation, Rizzoli Orthopaedic Institute, Bologna, Italy; 2 Institute of Science and Technology for Ceramics, National Research Council, Faenza, Ravenna, Italy; 3 Laboratory of Preclinical and Surgical Studies, Rizzoli Orthopaedic Institute, Bologna, Italy; 4 Laboratory of Biocompatibility, Innovative Technologies and Advanced Therapies, Rizzoli Orthopaedic Institute, Bologna, Italy; 5 Department of Biomedical Engineering, Columbia University, New York, New York, United States of America; University Hospital of Modena and Reggio Emilia, Italy

## Abstract

In case of degenerative disease or lesion, bone tissue replacement and regeneration is an important clinical goal. In particular, nowadays, critical size defects rely on the engineering of scaffolds that are 3D structural supports, allowing cellular infiltration and subsequent integration with the native tissue. Several ceramic hydroxyapatite (HA) scaffolds with high porosity and good osteointegration have been developed in the past few decades but they have not solved completely the problems related to bone defects. In the present study we have developed a novel porous ceramic composite made of HA that incorporates magnetite at three different ratios: HA/Mgn 95/5, HA/Mgn 90/10 and HA/Mgn 50/50. The scaffolds, consolidated by sintering at high temperature in a controlled atmosphere, have been analysed *in vitro* using human osteoblast-like cells. Results indicate high biocompatibility, similar to a commercially available HA bone graft, with no negative effects arising from the presence of magnetite or by the use of a static magnetic field. HA/Mgn 90/10 was shown to enhance cell proliferation at the early stage. Moreover, it has been implanted *in vivo* in a critical size lesion of the rabbit condyle and a good level of histocompatibility was observed. Such results identify this scaffold as particularly relevant for bone tissue regeneration and open new perspectives for the application of a magnetic field in a clinical setting of bone replacement, either for magnetic scaffold fixation or magnetic drug delivery.

## Introduction

Critical-sized bone defects are generally caused by trauma, bone diseases, prosthetic implant revision or tumor excision. The consequent bone tissue loss cannot be repaired by physiological regenerative processes. In these circumstances, current orthopaedic practice for critical-sized bone defects is to use autologous bone grafts, bone allografts, or synthetic graft materials. However, these strategies are unable to solve completely the problem, and motivate the development of novel orthopaedic strategies based on tissue engineering approaches to facilitate bone regeneration and repair [1], [2], [3]. In order to achieve this goal, scaffolds that mimic the three-dimensional (3D) tissue structure, are the most promising solutions. They must be biocompatible and osteoconductive with porosity ranging from 300–1000 µm diameter, necessary in supporting cell migration, proliferation, growth factor/nutrients diffusion [4], [5]. Porous bioceramic scaffolds have already demonstrated their excellent biocompatibility, bioactivity and osteoconductivity. In particular, hydroxyapatite (HA) based scaffolds are of significant interest since hydroxyapatite is the major inorganic component of natural bone [6]. In spite of this fact, none of the current HA scaffolds are able to entirely meet the demands of regenerative medicine strategies for bone repair. These treatments are unsuccessful especially for the case of large 3D scaffolds that lack rapid vascularisation. The ideal solution will enhance tissue regeneration and re-vascularisation with a continuous spatially-controlled delivery of cells and/or specific growth factors such as Vascular Endothelial Growth Factor (VEGF). In this way a more rapid and well-organized cell scaffold colonization will occur and it will be possible to control not only the timing but also the quality of the new tissue formation playing with the specific growth factors delivery.

Recently, the usage of magnetic nanoparticles (MNPs) for biological and medical purposes has been increasing and their biocompatibility is validated by several studies [7], [8], [9]. MNPs are unique in their reaction applications (e.g., hyperthermia, contrast agent for MRI, magnetic drug delivery and cell mechanosensitive receptor manipulation to induce cell differentiation), whereas only few authors have proposed approaches for their use in tissue engineering [10], [11], [12], [13], [14]. There are several limitations to the clinical application of a magnetic field for targeted therapy of a magnetic drug or for cell delivery. In fact, since the magnetic gradient decreases with the distance to the target, the main limitation of magnetic delivery relates to the strength of the external field that can be applied to obtain the necessary magnetic gradient to control the residence time of MNPs in the desired area or which triggers the drug desorption [15], [16]. The limitations inherent to the use of external magnetic fields can be circumvented by introduction of internal magnets located in the proximity of the target by minimally invasive surgery or by using a superparamagnetic scaffold under the influence of an externally applied magnetic field. In the latter, the magnetic moment of these scaffolds affords the potential for their continuous control and reloading with several tissue growth factors [17], [18]. The scaffolds will act effectively as a fixed “station” that provides long-term maintenance to the implanted tissue engineering constructs, providing the unique possibility to adjust the scaffold activity to the personal needs of the patient, overcoming the present difficulties of magnetic guiding. With this in mind, we have developed magnetic fully interconnected porous HA ceramic scaffold, by a ‘foaming’ technique in which different concentrations of magnetite nanoparticles were added during the synthesis. This study evaluates first the *in vitro* biocompatibility of these novel magnetic scaffolds in the presence or absence of a magnetic field, and afterwards the *in vivo* behavior of the most promising scaffold for clinical application in the repair of a bone defect.

## Materials and Methods

### Scaffold synthesis and characterization

The commercial HA powder (Finceramica Faenza Spa, Italy) was calcined at 1000°C for 2 hours in order to decrease the specific surface area value and prepare stable suspensions with higher solid loading. The optimized suspensions were prepared by ball mixing deionized water, 1.5 wt % of dispersant Dolapix CE-64 (Zschimmer & Schwarz, Lahnstein, Germany) and HA and Mgn powders having a specific surface area value of 3.93 m^2^/g and 10.41 m^2^/g, respectively. The following composite nominal compositions have been prepared: HA/Mgn weight ratio = 100/0, 95/5, 90/10 and 50/50. The HA/Mgn 100/0 scaffold represents the control group; this HA scaffold is already commercially available as a biomimetic bone graft (Engipore, Finceramica Faenza Spa, Italy). The free volume of the closed vessel used, compared to that occupied by the suspension, was set equal to 40% in volume. After 8 hours at 13 r.p.m, when an homogeneous slurry was obtained, 1.4 wt % of foaming agent (Dermocin BS, Fratelli Ricci, Italia) was added. After 12 hours the foamed slurry was casted in water draining moulds, allowing the setting of the foamed system and finally dried in air at room temperature for 48 hours. The thermal treatment and in particular the atmosphere conditions have been investigated so that to maintain high magnetization value (associated with the starting amount of magnetite powder) and to reach the highest consolidation extent. In particular, controlled atmosphere in Ar and of Ar/H_2_ were used up to a sintering temperature of 1200°C for 1 h. The volumetric density (apparent density) ρ of the foams was determined from the mass and dimensions of the sintered bodies. The porosity P was then calculated as P = 1−ρ/ρ_th_, with ρ_th_ corresponding to the theoretical density values of the HA/Mgn composites, calculated taking into account the theoretical value 3.16 g/cm^3^ for HA, 5.2 g/cm^3^ for magnetite and hematite and 4.9 g/cm^3^ for maghemite. Scanning electron microscopy (SEM; Stereoscan 360, Leica, Cambridge, UK) equipped with RX microprobe (EDS: INCA 300, Oxford Instruments, UK) was used for morphological and compositional evaluations. An optical microscope (Mic-D Digital Microscope, Olympus, Milano, Italy) was also used to investigate the pore sizes, window opening (pore interconnections) and strut thickness, measured with the help of an image analysis commercial software package (Image Pro-plus 4.5.1. Media Cybernetics, Silver Springs, MD). Magnetic measurements were performed at higher field in a superconducting quantum interference device (SQUID) magnetometer from Quantum Design (San Diego, CA, USA), capable of operating from 1.8 to 350 K under a maximum applied magnetic field of H = 7 N A**^−^** ^1^ m**^−^** ^1^.

### Cell culture and scaffold seeding

Saos-2 Human Osteoblast-like cells purchased by Lonza (Italy) were cultured in Dulbecco Modified Eagle's Medium (DMEM, PAA, Austria), containing penicillin/streptomycin (100 U/100 µg/ml) supplemented with 10% fetal bovine serum and kept at 37°C in an atmosphere of 5% CO_2_. Cells were detached from culture flasks by trypsinization and centrifuged; cell number and viability were assessed with trypan-blue dye exclusion test. Scaffolds were 6.00 mm diameter and 4.00 mm high, sterilized by 25 kGy γ-ray radiation prior to use. Scaffolds were placed one per well in a 24-multiwell plate well and pre-soaked in culture medium. Each scaffold was seeded by carefully dropping 50 µl of cell suspension (5×10^4^ cells) onto the scaffold upper surface, and allowing cell attachment for 1 h, before addition into each well of 1 mL of cell culture medium supplemented with 10 µg/ml ascorbic acid and 5 mM β-glycerophosphate for osteoblast activation. After a 6 h incubation step, each scaffold was carefully placed in a new 24 multiwell plate to eliminate any contribution of remnant cells from the cell suspension that might grow into the scaffold from its bottom surface. The medium was changed every 2 days. The experiments were conducted either with or without applying a magnetic field of 320 mT (MagnetoFACTOR-24, Chemicell, Germany) under the plates for each time point: 7 days, 14 days and 21 days, and osteoblast proliferation and activity in each scaffold analyzed. All cell handling procedures were performed in a sterile laminar flow hood. All cell culture incubation steps were performed at 37°C with 5% CO_2_.

### Cell proliferation assay

Total DNA content was quantified using the PicoGreen dsDNA (Invitrogen) assay following the manufacturer's suggested protocol. Briefly, five seeded scaffolds for each group per time point were transferred to a new multiwell plate and incubated with 1 ml 1× PBS with 0.1% (v/v) Triton-X for cell lysis. The sample was centrifuged at 11000 rpm for 1 min. 25 µL of supernatant was added to 175 µL of PicoGreen® reagent working solution in a 96-well plate. Fluorescence of the samples was measured with a microplate reader (Tecan, Research Triangle Park, NC) with excitation and emission wavelengths of 485 and 535 nm, respectively. The total number of cells in the sample was determined by converting the total DNA to cell number using the conversion factor of 7.7 pg DNA/cell [19]. Five samples were analysed per time point.

### Alkaline Phosphatase assay

Cell Alkaline Phosphatase (AP) activity was quantified using an enzymatic assay based on the hydrolysis of *p*-nitrophenyl phosphate (pNP-PO_4_) to *p*-nitrophenol (pNP) [20]. Briefly, samples were transferred to a new multiwell and incubated with 1 ml 1× PBS with 0.1% (v/v) Triton-X. The sample was centrifuged at 11000 rpm for 1 min. Supernatant was added to pNP-PO_4_ solution (Sigma-Aldrich) and allowed to react at 37°C. Absorbance was read at 0, 1, 2 and 3 min at λmax of 405 nm, using a Lambda 35 UV/VIS Spectrometer (PerkinElmer). AP activity was normalized to total cell number as measured by the Picogreen assay, at each time point AP activity was expressed as nanomoles of p-nitrophenol liberated per cell. Five samples were analysed per each time point.

### Live/Dead viability assay

Live/Dead viability assay was performed using a commercial kit, according to manufacturer's instructions (BioVision Research Products, Mountain View, CA, USA). Briefly, the Live/Dead solution was prepared by adding equal amounts of 1 mM Live-Dye and 2.5 mg/ml Propidium Iodide to the provided staining buffer. Samples were washed with 1× PBS for 5 min and incubated with the Live/Dead solution for 15 min at 37°C in the dark. Samples were rinsed three times in PBS, finely cut with a scalpel in order to examine also the internal surface, and images were acquired by an inverted Nikon Ti-E fluorescence microscope (Nikon). Three samples per time point were analysed.

### Actin staining

Samples were washed with 1× PBS for 5 min, fixed with 4% (w/v) paraformaldehyde for 15 min and washed with 1× PBS for 5 min. Permeabilization was performed with 1× PBS with 0.1% (v/v) Triton X-100 for 15 min. FITC-conjugated Phalloidin antibody (Invitrogen) 1∶500 in 1× PBS was added for 30 min at 37°C in the dark. Samples were washed with 1× PBS for 5 min and incubated with 300 nM DAPI solution (Invitrogen) for 5 min. Samples were washed with 1× PBS for 5 min and then finely cut with a scalpel in order to examine also the internal morphology. Analysis and imaging were performed by an Inverted Nikon Ti-E fluorescence microscope (Nikon). Three samples per time point were analysed.

### SEM characterization

Cell seeded scaffolds were imaged and characterized using a SEM Stereoscan 360 Scanning Electron Microscope (Cambridge Instruments, UK). Samples were washed with 0.1 M sodium cacodylate buffer pH 7.4 and fixed in 2.5% glutaraldehyde in 0.1 M sodium cacodylate buffer pH 7.4 for 2 h at 4°C, washed in 0.1 M sodium cacodylate buffer pH 7.4 and dehydrated in a graded series of ethanol for 10 min each. Dehydrated samples were finely cut with a scalpel in order to examine also the internal morphology, and then sputter-coated with gold using a Polaron Range sputter coater (DentonVaccum, USA) and mounted on a copper grid to be examined at SEM. One sample per time point was analysed.

### *In vivo* pilot experiment and histological analysis

The study was performed in accordance with EC guidelines (EC Council Directive 86/609, 1986) and the Italian legislation on animal experimentation (Decreto L. vo 116/92). The research protocol on animals has been approved by the Ethical Committee of Rizzoli Orthopaedic Institute and by the responsible public authorities. Six male rabbits (*Oryctolagus cuniculus*, Charles River, Lecco, Italy), 2.4±0.2 kg body weight, were housed at a controlled temperature of 22±1°C and relative humidity of 55±5% in single boxes and fed a standard diet (Mucedola, Milano, Italy) with filtered tap water *ad libitum*. After quarantine of at least 10 days, the animals were fasted for 24 hours before surgery. The animals were subjected to surgery to implant the tested scaffolds at the distal femoral epiphysis under general anaesthesia and in aseptic conditions. After having shaved and disinfected the posterior legs, the animals underwent a lateral longitudinal incision of lateral femoral condyle. Femoral lateral condyle trabecular bone was cross-sectionally drilled at low speed and a profuse irrigation with cold sterile 0.9% NaCl solution was maintained throughout the process to prevent the risk of bone necrosis. A critical bone defect of 6.00 mm in diameter and 8.00 mm in depth was made in each lateral femoral condyle. All six animals were subjected to the implantation of one HA/Mgn 90/10 scaffold and one HA scaffold was implanted in the contralateral condyle as a control group. Scaffolds were 6.00 mm diameter and 8.00 mm high, sterilized by 25 kGy γ-ray radiation. Finally, the skin was sutured. General anaesthesia was induced by an intramuscular injection of 44 mg/kg ketamine (Imalgene 1000, Merial Italia S.p.A, Milan, Italy) and 3 mg/kg xylazine (Rompun, Bayer SpA, Milano, Italy) under assisted ventilation with O_2_/N_2_O (1/0.4 l/min) mixture and 2.5% isofluorane (Forane, Abbot SpA, Latina, Italy).

Post-operatively, antibiotics and analgesics were administered: 0.6 ml/kg flumequil (Flumexil, (FATRO SpA, Bologna, Italy) and 0.1 ml/kg/day metamizole sodium (Farmolisina, Ceva Vetem SpA, Monza-Brianza, Italy).

At 4 weeks after surgery, the animals were pharmacologically euthanized with intravenous administration of Tanax (Hoechst, Frankfurt am Main, Germany), under general anaesthesia. The operated bone segments were excised and stripped of soft tissue and the presence of haematomas, oedema, and inflammatory tissue reactions were macroscopically evaluated. The bone segments were fixed in 4% buffered paraformaldehyde for 24 hours, dehydrated in a graded series of alcohol and finally embedded in a methyl methacrylate resin (Merck Schuchardt OHG, Hohenbrunn, Germany). Using a saw microtome (Leica SP1600, Leica Microsystems Srl, Italy), three consecutive central sections to the major axis of the implant for each bone segment were cut (60±20 µm) and polished (Struers Dap-7, Struers Tech A/S, Rodovre/Copenaghen, Denmark). Then, thinned sections (30±10 µm) were stained with Toluidine Blue, Acid Fucsin and Fast Green.

### Statistical analysis

For cell proliferation and AP activity, results were expressed as MEAN±SEM plotted on graph (n = 5). Statistical analysis was performed using two-way ANOVA, followed by Bonferroni's post-hoc test, for the analysis of magnetic field effect, time effect and magnetic field versus time effect. Analysis of differences between groups, for each time point, was performed by one-way ANOVA, followed by Tukey's post-hoc test. All statistical analysis made use of the GraphPad Prism software (version 5.0), with α = 0.05.

## Results

No undesired secondary phases were detected by XRD in the sintered magnetic composites, beside hydroxyapatite and magnetite: the starting nominal composition was maintained up to 10 wt% of magnetite. For higher magnetite content abouot 20 wt% of hematite was detected as secondary phase. The sintered composites showed a total porosity ranging between 73 to 78%. As observed by SEM, the microstructure of the sintered samples is characterized by large pores in the range 300–800 µm uniformly distributed throughout the sample with numerous interconnections (average dimension = 100–150 µm) between the individual macropores. Moreover SEM/EDS investigation showed that the magnetic phase is uniformly distributed and merged in the calcium phosphate matrix. At high magnetic field (7 N A**^−^** ^1^ m**^−^** ^1^) magnetization showed values of 34 and 53 emu/g for the samples containing 5 wt% and 10 wt% of magnetite respectively; while dropped at 37 emu/g in case of the composite containing 50 wt% of magnetite due to the presence of hematite as secondary phase.

Here, we analyzed effects of the addition of magnetite for bone tissue regeneration *in vitro*, using Human osteoblast-like cells and *in vivo* on a critical lesion of the rabbit femoral condyle as a pilot study.

The *in vitro* investigation was performed on cell-seeded scaffolds, where cells were seeded onto the scaffold surface. All the experiments were conducted with or without applying a 320 mT magnetic field and the analysis of osteoblast proliferation and activity in each scaffold has been performed at 7, 14 and 21 days post-seeding.

**Figure 1:**
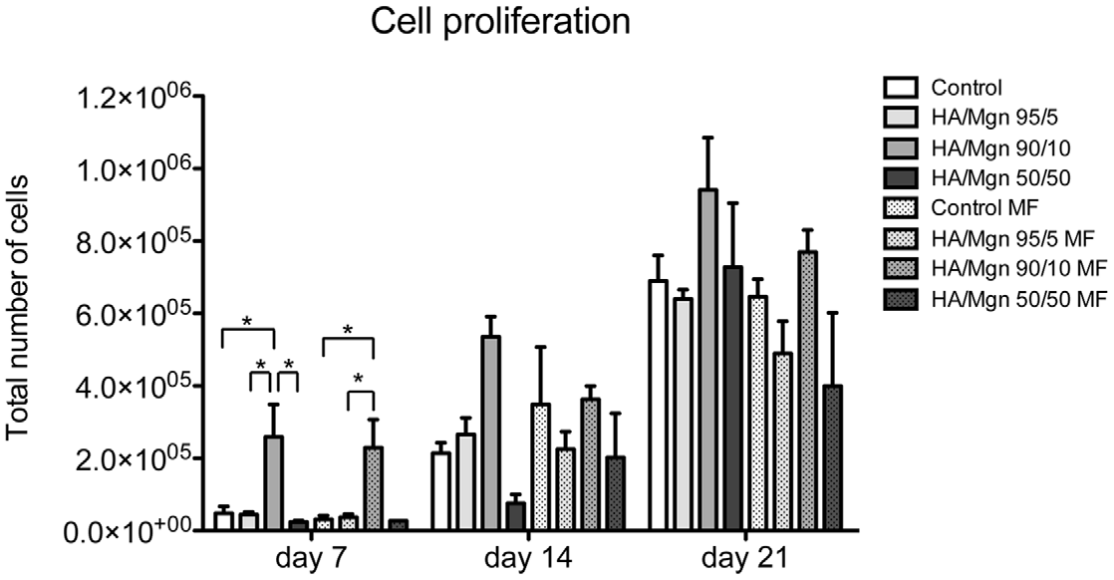
Cell proliferation assay. The Picogreen DNA content assay was performed on cultures of osteoblast-like cells seeded on different HA/Mgn scaffolds at 7, 14 and 21 days of culture, either in the presence or absence of a magnetic field (MF) (n = 5). HA porous scaffold is used as control group. * p≤0.05.

We have observed an overall increase in cell proliferation from day 7 to day 21 for all groups. Moreover, we have found a statistical significant increase in cells seeded on the HA/Mgn 90/10 after 7 days, both with or without a magnetic field (Fig. 1). At day 14, the group HA/Mgn 90/10 continued to have higher cell proliferation respect to the other groups, with this trend maintained until the end of the experiment (Fig. 1).

**Figure 2:**
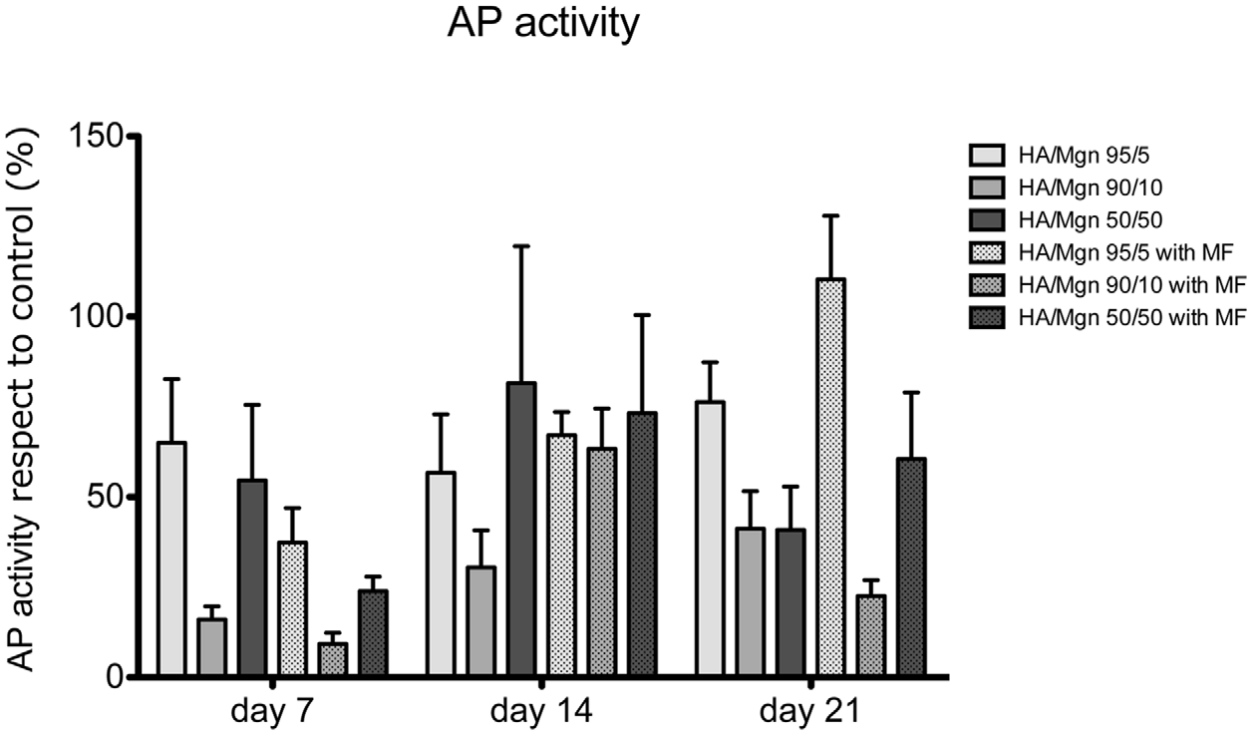
Alkaline phosphatase activity assay. AP activity was measured on different HA/Mgn scaffolds seeded with human osteoblast-like cells at 7, 14 and 21 days, either in the presence or absence of a magnetic field (MF) (n = 5). Results are normalized to control.

Alkaline phosphatase activity, an osteogenic marker, was analysed and results normalized with the control. No statistically significant differences were found between experimental groups, and the applied magnetic field did not affect the cellular alkaline phosphatase activity (Fig. 2).

**Figure 3:**
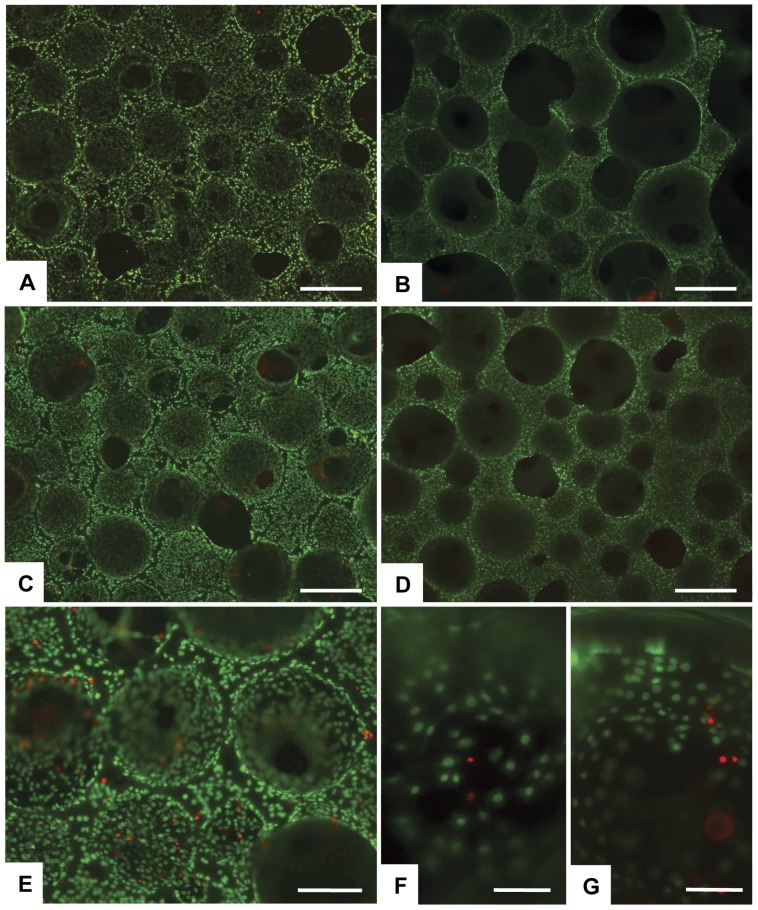
Analysis of cell viability. Cell viability was analysed by the Live/Dead assay (n = 3). Live-Dye stains for live cells in green, Propidium Iodide stains for dead cells in red. A) Control at day 7. B) HA/Mgn 95/5 at day 7. C) HA/Mgn 90/10 at day 7. D) HA/Mgn 50/50 at day 7. E) HA/Mgn 95/5 at day 14. F) HA/Mgn 95/5 at day 14. G) HA/Mgn 50/50 at day 14 with applied magnetic field. Scale bars: A–D) 500 µm. E) 250 µm. F, G) 100 µm.

The Live/Dead assay showed a very high ratio of viable cells at each experimental time point, with a range between 73.9±4.3% and 92.8±3.0% and no differences between groups. No significant effect of magnetic field application was observed. After 7 days of culture, cells cover nearly the entire upper scaffold surface (Fig. 3A–D). Cells grew into the porous scaffold structure and infiltrated the scaffolds as shown in Fig. 3E. The very small ratio of dead cells found on magnetic materials was very similar to that found in the HA group for all time points (Fig. 3A–E). We analyzed also the inner surface after cutting the samples. After 14 days, cells had infiltrated into the scaffold and a high number of live cells were seen in all groups (Fig. 3F, G).

**Figure 4:**
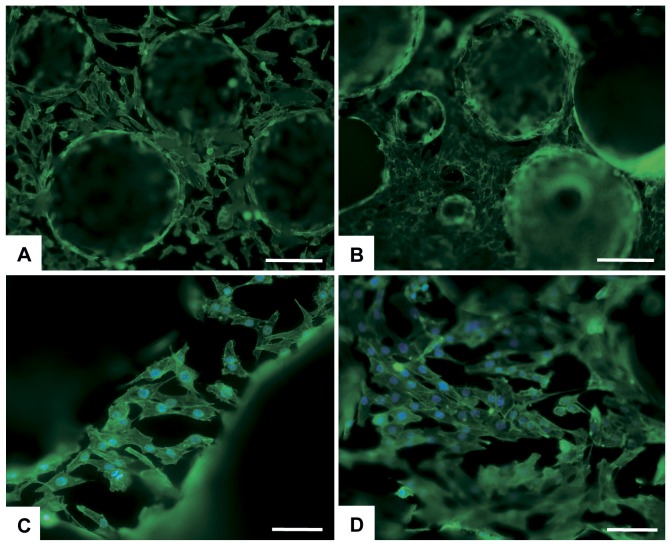
Analysis of cell morphology. Cell morphology was analysed by actin staining (n = 3). Actin is shown in green, DAPI in blue. A) Control at day 14. B) HA/Mgn 90/10 at day 14. C) Control at day 21 with applied magnetic field. D) HA/Mgn 90/10 at day 21 with applied magnetic field. Scale bars: A, B) 250 µm. C, D) 100 µm.

Analysis of phalloidin staining on day 7, 14 and 21 after seeding did not reveal differences in cell morphology between groups, in the presence or absence of a magnetic field. Attached cells exhibited their characteristic intricate morphology both on the external and inner surface (Fig. 4 A–D).

**Figure 5:**
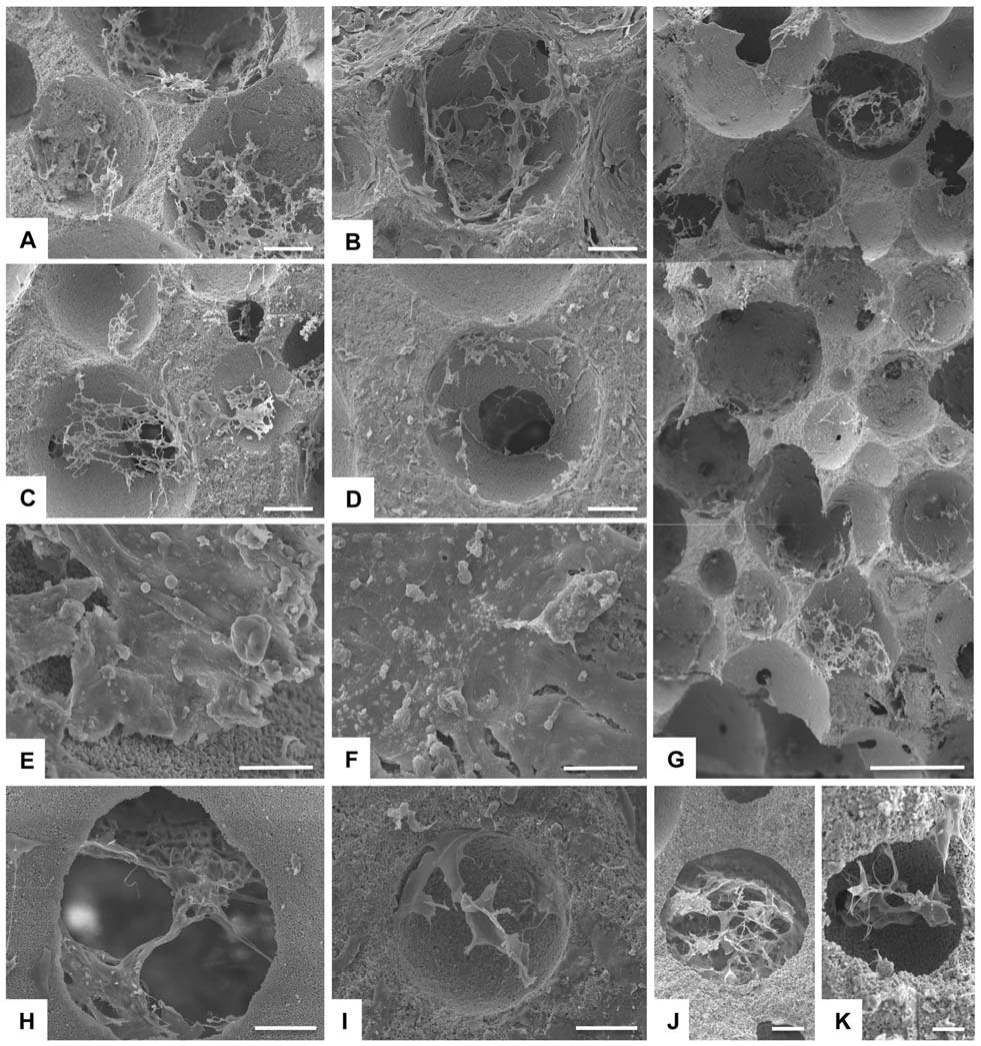
SEM characterization of cell seeded scaffolds. Detailed analysis of cell morphology was assessed by scanning electron microscopy. A) Control at day 7. B) HA/Mgn 95/5 at day 7. C) HA/Mgn 90/10 at day 7. D) HA/Mgn 50/50 at day 7. E) Control at day 14 with applied magnetic field. F) HA/Mgn 90/10 at day 14 with applied magnetic field. G) HA/Mgn 95/5 at day 7, vertical overlapping profile of 3 consecutive images. H) Control at day 7. I, J) HA/Mgn 90/10 at day 7. K) HA/Mgn 95/5 at day 7. Scale bars: A–D) 150 µm, E, F) 10 µm, G) 500 µm, H, I) 50 µm, J) 50 µm, K) 20 µm.

Detailed cell morphology was analysed by scanning electron microscopy. After 7 days, cells almost covered the external surface of the entire scaffold, and infiltrated into the pores (Fig. 5A–D).

Significant integration of cells to the scaffold was also confirmed by SEM images. In fact after 14 days cells were firmly attached the to scaffold surface and the scaffold external surface was completely embedded by a cell layer and no differences were found between groups (Fig. 5 E, F). The inner section of each scaffold showed that after 7 days cells had already entered the pores and attached to the inner surface, as shown by the section reconstruction in Fig. 5G. Cells seeded onto the upper surface after 7 days migrated through the interconnected porous structure and cells were seen well attached to the lower level of the scaffold (Fig. 5G).

Looking at the details, cells not only grew inside the pores, but they also nicely bridged the porous structure forming a cell network (Fig. 5 H–K).

Overall, *in vitro* results showed that there were no differences in cell viability and cell morphology between groups, but the quantification of DNA demonstrated that the HA/Mgn 90/10 significantly increase the cell proliferation, in particular in the first 7 days. For this reason, we selected this scaffold for a pilot experiment *in vivo*.

**Figure 6:**
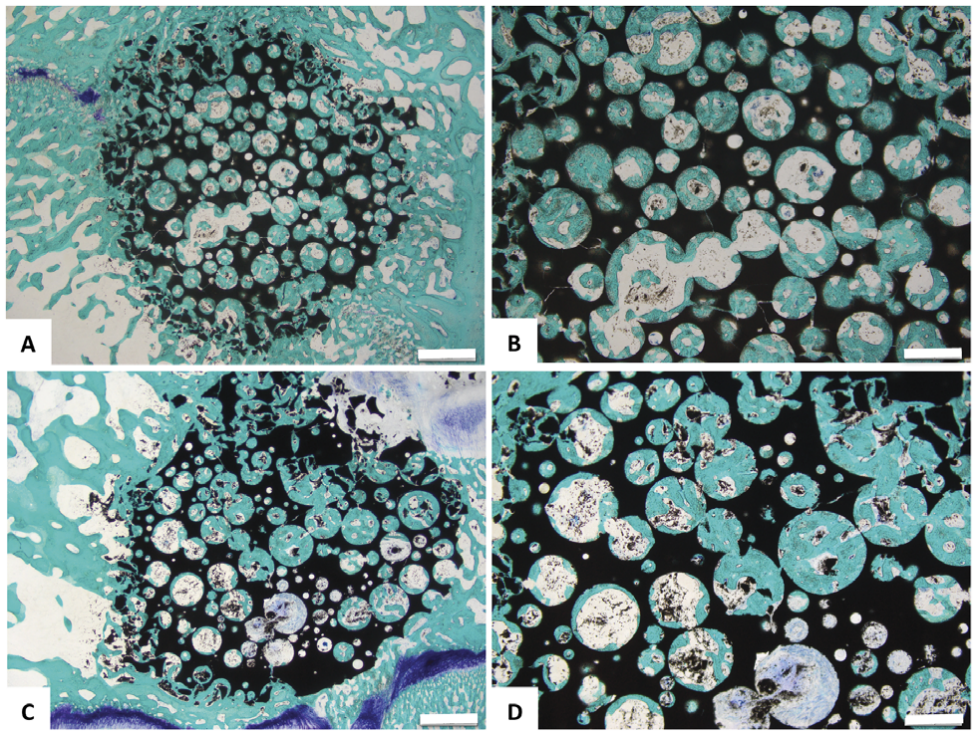
Histological evaluation of the *in vivo* implanted scaffolds. Toluidine Blue, Acid Fucsin and Fast Green staining shows similar histocompatibility for both scaffolds 4 weeks after implantation (n = 6). A, B) Control, C, D) HA/Mgn 90/10. Scale bars: A, C) 1 mm, B, D) 500 µm.

At 4 weeks post-implantation, macroscopic evaluation showed the HA porous implants to be in the proper position and there was no evidence of haematoma, oedema, infection or tissue necrosis in either bone and peri-implant soft tissue associated with control or magnetic implants. Bone tissue was well visible around and inside the scaffold in both groups (Fig. 6A, C). Due to the interconnected porous structure, bone regenerated into the magnetic scaffold and after only 4 weeks some pores were completely full of new bone proving a good level of histocompatibility of the scaffold comparable to the control group (Fig. 6B, D).

## Discussion

The spatial and physical properties as well as biochemistry of biomaterials strongly influence cell growth into a scaffold. It is well known that a highly porous scaffold is desirable in tissue engineering, since it can support the necessary nutrient transport for tissue regeneration [21], [22]. Porosity also plays an important role in cell migration whereas pore interconnectivity increases the overall surface area for cell attachment and facilitates cell ingrowth into the scaffolds. Notwithstanding the fact that many scaffolds with these features were developed in the last few decades to treat critical bone defects, none have been adopted for routine treatment of bone defects in the clinic.

The magnetic scaffolds presented in this study were well chemically characterized and exhibited good biocompatibility, as assessed previously (manuscript in preparation). Here, 3D cell cultures were investigated in order to better understand which scaffold would promote more rapid cell scaffold colonization and subsequent tissue regeneration in *in vivo* applications. The *in vitro* results showed good cell distribution and cell morphology in the range of magnetic HA scaffolds studied, comparable to the magnetite-free control scaffold already used clinically for bone repair. The application of magnetic field did not adversely affect cell morphology or alkaline phosphatase activity, indicating that higher magnetization can be used for prosthesis fixation purposes or for guidance without impairing bone tissue regeneration.

With respect to osteoblast performance on each scaffold, a significant difference was found in cell proliferation between the HA/Mgn 90/10 scaffold and the remaining groups, although AP activity did not significantly increase, reflecting an osteoblast commitment towards proliferation. Cell number was considerably higher in particular in the first week after seeding, therefore the HA/Mgn 90/10 scaffold has the potential to strongly enhance *in vivo* tissue regeneration at an early stage.

There is debate regarding the use of magnetite in medical applications. There are studies that demonstrate the toxic effect of magnetite on cell culture, but at the same time there are several works that show the opposite result. In particular, if we focus on the magnetite use for scaffold in musculoskeletal application, there are numerous studies with good results both with stem cells or cell lines which are in accordance with our results [23], [24], [25], [26].

For the above-mentioned reasons, the HA/Mgn 90/10 construct was evaluated in a critical-sized defect of the rabbit femoral condyle as a pilot experiment. After 4 weeks, the HA/Mgn 90/10 construct showed mineralized tissue regeneration into its structure in a similar manner as the control non-magnetized HA construct.

The optimal tissue regeneration process, in addition to scaffold properties, depends on a complex cascade of biological events controlled by an interplay of cytokines and growth factors. These provide local signals at sites of injury, which regulate the mechanisms and pathways that govern tissue regeneration [27], [28]. For instance, the creation of a vascular network assuring diffusion of nutrients and removal of metabolites over long distances are critical for guiding osteogenesis. As such, insufficient activation of signalling molecules and, in particular, the generation of a poor vascular network often impedes new bone formation [29], [30].

The encouraging results obtained *in vivo* with the HA/Mgn 90/10 scaffold open future prospects in bone tissue engineering and also for the use of an applied magnetic field for various clinical applications. In fact, under an external magnetic field, this scaffold can be activated and function like a magnet, attracting functionalized magnetic nanoparticles injected close to the scaffold. Using this approach, strategies for drug delivery using nanoparticles could be made more effective by reducing nanoparticle loss [15], [16]. Thus, there is potential to enhance tissue regeneration via delivery of several growth factors that can be accurately released close to or into the scaffold. Magnetic scaffolds could provide a novel and simple solution to the general problem of orthopaedic device fixation [31], [32]. We envision that efficient scaffold fixation *via* magnetic forces could be achieved by magnetization of the scaffold at a level sufficient to activate an attractive force towards selected anchoring magnetic objects.

Improved outcomes associated with the use of engineered orthopaedic tissues will depend upon an optimized scaffold design that provides 3D structural support, allows initial cellular transport and promotes subsequent integration with native tissue. These aspects are likely to be enhanced by modifications that improve the ability of scaffolds to deliver growth factors at therapeutic levels during tissue regeneration. The development and use of magnetic scaffolds, such as those described in this study, in conjunction with magnetic drug delivery constitute a very innovative and promising technology to treat not only musculoskeletal defects but several pathologies in the area of regenerative medicine.
